# Deformable Nanovesicles Synthesized through an Adaptable Microfluidic Platform for Enhanced Localized Transdermal Drug Delivery

**DOI:** 10.1155/2017/4759839

**Published:** 2017-04-05

**Authors:** Naren Subbiah, Jesus Campagna, Patricia Spilman, Mohammad Parvez Alam, Shivani Sharma, Akishige Hokugo, Ichiro Nishimura, Varghese John

**Affiliations:** ^1^Drug Discovery Lab, Department of Neurology, University of California, Los Angeles, CA, USA; ^2^Weintraub Center for Reconstructive Biotechnology, School of Dentistry, University of California, Los Angeles, CA, USA; ^3^California NanoSystems Institute, University of California, Los Angeles, CA, USA; ^4^Regenerative Bioengineering and Repair Laboratory, Department of Surgery, University of California, Los Angeles, CA, USA

## Abstract

Phospholipid-based deformable nanovesicles (DNVs) that have flexibility in shape offer an adaptable and facile method to encapsulate diverse classes of therapeutics and facilitate localized transdermal delivery while minimizing systemic exposure. Here we report the use of a microfluidic reactor for the synthesis of DNVs and show that alteration of input parameters such as flow speeds as well as molar and flow rate ratios increases entrapment efficiency of drugs and allows fine-tuning of DNV size, elasticity, and surface charge. To determine the ability of DNV-encapsulated drug to be delivered transdermally to a local site, we synthesized, characterized, and tested DNVs carrying the fluorescently labeled hydrophilic bisphosphonate drug AF-647 zoledronate (AF647-Zol). AF647-Zol DNVs were lyophilized, resuspended, and applied topically as a paste to the calvarial skin of mice. High-resolution fluorescent imaging and confocal microscopy revealed significant increase of encapsulated payload delivery to the target tissue—cranial bone—by DNVs as compared to nondeformable nanovesicles (NVs) or aqueous drug solutions. Interestingly, NV delivery was not superior to aqueous drug solution. Our studies show that microfluidic reactor-synthesized DNVs can be produced in good yield, with high encapsulation efficiency, reproducibility, and stability after storage, and represent a useful vehicle for localized transdermal drug delivery.

## 1. Introduction

Transdermal application has numerous advantages as a route for drug delivery, including—when patch application is used—continuous delivery which results in stable drug levels, ease of application, and elimination of the need for dosage schedule adherence; perhaps most importantly, transdermal delivery has resulted in a reduction in organ toxicity for some drugs used chronically [1, 2]. The broad use of transdermal delivery, however, has been limited by the requirements for very specific physiochemical properties of the drug to be delivered. However, recent advancements in transdermal delivery including the use of microneedles [3], iontophoresis [4], biopolymers/biomaterials [5, 6], and encapsulation of drugs in liposomes—small (nano)synthetic vesicles comprising a lipid bilayer (nanovesicles, NV)—have allowed progress. Development of “elastic” deformable nanovesicles (DNVs) [7, 8] in particular has been key to this progress.

Transdermal delivery, by definition, requires drug passage through the skin. Skin is an excellent protective barrier, adept at preventing the entry of foreign substances, ultraviolet radiation, and microbial or viral pathogens to the body [9], a difficult barricade to breach for drug delivery. A key feature of this barricade is the stratum corneum, a layer of tightly packed, keratinized dead cells forming the outermost component of the epidermis, which is about 10–40 *μ*m thick [10]. The only ingress across this layer is through nanopore lacunae, which are approximately 50 nm or smaller, and even then requires facilitation to allow drug entry [11]. Conventional NVs over this ~50 nm size fail to penetrate the stratum corneum and are thus excluded from the deeper layers of the skin [12]. As postulated by Cevc [13], DNVs synthesized with surfactant edge activators are predicted to be elastic enough to deform and squeeze through these nanopores without rupturing and leaking their payload into the systemic circulation. It was further postulated that hydration and osmotic forces were driving these nanoparticles, which seek the deeper layers of the skin for hydration. Cevc's transfersomes® and other subsequently developed deformable vesicles such as ethosomes [7] (liposomes with a high concentration of ethanol) have shown success in transdermal delivery, primarily for certain lipophilic substances.

The conventional NV and DNV production methods are often based around hand-mixing and postprocessing involving sonication and/or extrusion and freeze-thawing. It has been recognized that commonly used postprocessing methods often compromise membrane integrity and composition and thus are unsuitable for use in encapsulation of degradation-sensitive therapeutics. These methods have also failed to be successful in encapsulating drugs of various classes and solubilities in a defined, predictable manner. Conventional synthesis methods are burdened with a lack of reproducibility in vesicle size and drug entrapment efficiency, which have critically hindered the clinical development of NVs and DNVs [14–17].

A microfluidic-based NV production method may prove to be a highly effective remedy for these technical issues. Leaders in this burgeoning field include Jahn et al. [18, 19] and Hood et al. [20, 21], who published recent studies on microfluidic-enabled NV synthesis, hydrodynamic focusing, and associated concepts. Within the narrow channels of a microfluidic reactor chip, various membrane components dissolved in specified ratios in organic and aqueous streams as appropriate, at precisely controlled speeds and ratios, are allowed to mix diffusively. The microscale dimensions of these confined channels restrict turbulent flow, resulting in well-defined mixing and homogenous, reproducible NV populations. Moreover, the laminar flow patterns of the microchannels can be predicted accurately and subjected to mathematical modeling allowing for the manipulation of micropatterned surface design. As the faster flowing aqueous stream mixes with the organic stream, phospholipids are thrown into an aqueous medium and assemble to form unilamellar vesicles to establish thermodynamic stability and in the process entrap any accompanying payload dissolved in the streams. This powerful technique renders possible encapsulation of a wide variety of drug classes, including RNA, DNA, proteins, and both hydrophobic and hydrophilic small molecules, without relying on destructive postprocessing techniques to control size and lamerality.

In this study, we developed an adaptable synthesis methodology for a DNV-based system using a commercially available microfluidic reactor. Though the microfluidic reactor chip used in this study was fabricated by and purchased from a company, the base technology is simple and is being used by an ever-expanding number of research groups [22, 23]. In our application, we present the encapsulation in NVs and DNVs of a hydrophilic model drug, a fluorescently tagged bisphosphonate—AF647-zoledronate (AF647-Zol)—with high efficiency, utilizing a modified method of microfluidic synthesis. We further expanded on prior DNV studies which focused their investigations on transdermal penetration* in vitro* via Franz diffusion cell experiments [24] to* in vivo* investigations in murine models. Our experimental results show that cargo-loaded DNVs can successfully deliver AF647-Zol through the skin barrier to an underlying target tissue, in this case, bone. A delivery system capable of such a task could reduce the amount of drug required for treatment and have considerable implications in a number of fields (albeit with different tissue targets), including dentistry [25], pain management [26], wound healing [27], cosmeceuticals, hair regrowth [28], tattoo removal [29], lymph node targeting, and Langerhans cell-mediated immunotherapy [30].

## 2. Materials and Methods

### 2.1. Preparation of a Dried Lipid Mixture

A dried lipid mixture was synthesized by combining 1,2-dipalmitoyl-sn-glycero-3-phosphocholine (DPPC; Sigma Aldrich, St. Louis, MO, USA), cholesterol (Sigma Aldrich, St. Louis, MO), and N-[1-(2,3-dioleoyloxy)propyl]-N,N,N-trimethylammonium methyl-sulfate (DOTAP; Avanti Polar Lipids, Alabaster, AL, USA) as 10 mM solutions in chloroform (Sigma Aldrich, St. Louis, MO, USA) in a 5 : 3 : 2 volume ratio. DOTAP is a cationic lipid commonly used as a cellular transfection agent for nucleic acid delivery. Other lipid components tested included the neutral lipid 1,2-dioleoyl-sn-glycero-3-phosphoethanolamine (DOPE; Sigma Aldrich, St. Louis, MO, USA) or negatively charged diacetyl phosphate (DCP; Sigma Aldrich, St. Louis, MO, USA) in place of DOTAP, with DPPC and cholesterol in the same 5 : 3 : 2 ratio. Chloroform was chosen as an initial step solvent due to its high solubility potential for various lipid classes. The mixture was allowed to dry by solvent evaporation under rotation for 5 minutes in a Rotovap (Heidolph) or under ventilation for 48 hours.

### 2.2. Input Stream Preparation

#### 2.2.1. Organic

The dried lipid mixture was resuspended and dissolved in isopropyl alcohol (Sigma Aldrich, St. Louis, MO, USA) in a volume equivalent to the chloroform initially used before drying to maintain a lipid mix concentration of 10 mM.

#### 2.2.2. Aqueous

The aqueous stream comprised deionized water filtered through a 0.22 *μ*m membrane Steriflip vacuum setup (Millipore, Billerica, MA, USA). For compound/drug loading, fluorescein isothiocyanate (FITC; ThermoFischer Scientific, Waltham, MA, USA) or AF647-Zoledronate (BioVinc LLC, Culver City, CA, USA) was loaded at a concentration of 10–70 nM; the AF647-Zol concentration was determined based on earlier studies involving intravenous injection of AF647-Zol in animal models [31, 32].

### 2.3. Microfluidic Reactor Setup

A Syrris microfluidic reactor system (Royston, UK) was used for NV/DNV synthesis. There are two input streams, here an aqueous one and an organic one, each connected to its own pump. These two pumps are connected to two separate liquid storage containers of variable volumes, termed “loops.” The system is running when setup is changed from “Fill” to “Inject”; the contents of the loop are directed through microchannels to the 26 *μ*L reactor chip and exit through an outlet to a collection tube. For DNV and NV synthesis, the membrane components are dissolved in the organic stream and drug is dissolved in the appropriate stream according to its solubility. Depending primarily on the choice of drug to be loaded, one of two synthesis strategies may be employed: the standard approach or the modified method. For AF647-Zol, the modified method was used.

#### 2.3.1. Standard Method: Low Speed and High Flow Rate Ratio

The aqueous stream was pumped to the reactor at 1000 *μ*L/min and the organic stream was pumped to the reactor at 10 *μ*L/min, for a resulting flow rate ratio of 100.

#### 2.3.2. Modified Method: High Absolute Speed and Low Flow Rate Ratio

The aqueous stream was pumped to the reactor at 5000 *μ*L/min and the organic stream was pumped to the reactor at 1000 *μ*L/min, for a resulting flow rate ratio of 5. Flow rate ratios of 7 and 10 were also tested, by setting the aqueous stream to 7000 *μ*L/min and 10,000 *μ*L/min, respectively.

### 2.4. Characterization

*(i) Size and Dispersity*. Samples of reactor-synthesized NV/DNVs were diluted 10x and 100x in deionized water and analyzed by the technique of dynamic light scattering on a Malvern Zetasizer (Nano-ZS; Malvern, Worcestershire, UK) at 25°C. Three measurements, each averaging a hundred runs, were performed on each sample, reported here as mean ± standard deviation.

*(ii) Zeta Potential*. The surface charge of NV/DNV formulations was assessed by zeta potential measurements by dynamic light scattering under electrophoresis on a Malvern Zetasizer (Nano-ZS: Malvern, Worcestershire, UK) at 25°C. Samples were diluted in 10x and 100x deionized water. Three measurements, each averaging a hundred runs, were performed on each sample, reported here as mean ± standard deviation. 

*(iii) Entrapment Efficiency*. Upon collection from the reactor, samples were dialyzed in a G2 dialysis cassette (ThermoFisher Scientific, Waltham, MA, USA) for 24 hours in a 1000-fold dialysis volume. The dialysis solution was replaced at 12 h and 18 h. The dialyzed samples were probe sonicated extensively for prolonged periods of time (Probe Solicitor; Manufacturer) to rupture vesicle membranes and release the content. The drug content of the ruptured sample was quantified by fluorescent spectroscopy (for FITC and AF647-Zol). 

*(iv) AFM Analysis*. All atomic force microscopic (AFM) measurements of the nanovesicles were done under ambient conditions after absorption on a mica surface using a Dimension Icon AFM (Bruker Corporation, CA). AFM measurements were collected in tapping mode using silicon cantilevers (TESPW, Bruker) at 1 Hz and 256 samples/line. The height, amplitude, and phase were obtained for the AF647-Zol DNVs and conventional NVs. Images were plane fit using Nanoscope software (version 9.0).

#### 2.4.1. Morphology by Transmission Electron Microscopy

Samples of NVs and DNVs loaded with AF647-Zol produced by the modified microfluidic method were diluted 500x, absorbed onto a copper mesh (EMScience, Cat #FCF400-Cu) for 18 minutes, and fixed with glutaraldehyde for 3 minutes. Following a wash, they were stained with uranyl acetate solution for 3 minutes. They were then imaged on a JEOL 100CX electron microscope at 58,000 to 100,000 times magnification.

#### 2.4.2. Confocal Microscopy

Samples of DNVs loaded with AF647-Zol produced by the modified microfluidic method were dialyzed and lyophilized as described below. The lyophilized samples were then resuspended to a 100x dilution of their original postsynthesis concentration. Resuspended samples were imaged in fluid on a glass slide through an SP5 Leica Confocal Microscope (Leica Microsystems, Waltzer, Germany) to determine postlyophilization vesicle viability and drug leakage.

### 2.5. Storage and Preparation for* In Vivo* Application: Dialysis, Lyophilization, and Resuspension

Upon collection from the reactor, samples were dialyzed in a G2 dialysis cassette (ThermoFisher Scientific, Waltham, MA, USA) for 24 hours in a 1000-fold dialysis volume. The dialysis solution was replaced at 12 h and 18 h. The NVs/DNVs were then lyophilized (Labconco, Kansas City, MO) for 24 hours until they were converted to a powder. For* in vivo* application to murine calvarial skin, the lyophilized formulations were resuspended in 0.9% saline to a thick liquid paste-like consistency, concentrated 150-fold from postsynthesis concentration.

### 2.6. *In Vivo* Trial Experimental Design

Thirteen C57Bl6J wildtype mice were divided into three groups of four representing topical treatment with (i) AF647-Zol-loaded DNV paste, (ii) AF647-Zol-loaded NV paste, or (iii) aqueous solution of AF647-Zol, with the remaining mouse receiving only 0.9% saline as a negative control.

Prior to topical application, each of the thirteen mice had the hair on their calvarial skin shaved by a maxillofacial surgeon with great care taken to prevent injury or scratches. The mice were then anesthetized by isoflurane inhalation and the appropriate formulation was applied topically and unoccluded on the calvarial skin above the skull and spread in a square pattern. The mice remained anesthetized for an hour following application to prevent grooming-related drug removal. Mice were sacrificed 48 hours later by CO_2_ inhalation followed by cervical dislocation, and their skull, calvarial skin, and (right or left) femur were extracted and analyzed through several fluorescent analysis techniques to ascertain the efficacy of penetration and drug delivery. All animal experiments were performed with a protocol approved by the Animal Research Oversight Committee and in accordance with all the rules and regulations for animal use and care at UCLA.

### 2.7. Tissue Dissection and Analysis

The extracted skull, femur, and calvarial skin surface were imaged by an LAS3000 luminescent image analyzer (Fujifilm, Tokyo, Japan). In each skull image, the standardized circular area centered by the intersection between the coronal suture and the sagittal suture was determined as the region of interest (ROI). The total AF647 intensity in the ROI was measured using an open source, Java-based image processing program (ImageJ, https://imagej.nih.gov/ij/, NIH, Bethesda, MD). The mean and standard deviation were calculated in each group and compared by Dunnett's test. The statistical significance was reached at *p* < 0.05.

Portions of the extracted skin and skull bone were fixed in 70% ethanol and sectioned and stained with hematoxylin and eosin to analyze histology. The skin and skull bone were also cryosectioned and mounted with a fluorescence-protective media with DAPI. These cryosectioned samples were analyzed by confocal laser scanning microscopy to discern the depth and degree to which the different formulations permeated through the layers of the skin and deposited the payload to the underlying target site.

## 3. Results and Discussion

### 3.1. Formulation of NVs and DNVs

The method parameters used here for NV/DNVs were chosen through rational consideration of the components, review of existing literature, and testing. 1,2-Dipalmitoyl-sn-glycero-3-phosphocholine (DPPC), a tried and tested phospholipid that is inexpensive and readily available, was the primary component of the lipid mixture at 50 mol% (w/w). Cholesterol was chosen as the second component of the lipid mixture at 30 mol% (w/w), as it is an integral component of biological membranes and a well-known regulator of membrane fluidity and rigidity at physiological temperature and pH. To control the surface charge, we chose a positively charged N-[1-(2,3-dioleoyloxy)propyl]-N,N,N-trimethylammonium methyl-sulfate (DOTAP), negatively charged diacetyl phosphate (DCP), and neutral 1,2-dioleoyl-sn-glycero-3-phosphoethanolamine (DOPE), to make up the remainder of the lipid mixture.

The lipid mixture described above was exactly the same for both NVs and DNVs. For the synthesis of DNVs, the only difference is the addition of an edge activator to the above lipid mixture. For this purpose, sorbitan monooleate (Span 80), a nonionic surfactant that is a GRAS material, was added to the mixture at 15% (w/w), which was previously reported to be an optimum edge activator/concentration for skin penetration in* in vitro* Franz diffusion cell experiments [24]. Span 80 was chosen based on its biocompatibility and low HLB number (~3.4), which allows it to intercalate well within the membrane and exert its surface tension-reducing properties, thus destabilizing the membrane and increasing its elasticity.

### 3.2. Salient Properties of Microfluidic Reactor-Produced NVs and DNVs

#### 3.2.1. Microfluidics Reaction Principle

When phospholipid molecules are dispersed in aqueous solution, thermodynamic forces induce them to self-assemble into aggregates to minimize the Gibb's free energy. Due to their amphiphilic structure, this stable aggregate is vesicular and is either micellar or bilayered. In microfluidic production, lipids contained in a slow-flowing organic solvent (isopropanol for example) that is miscible with water are mixed with a much faster flowing aqueous solution and thus are forced to quickly aggregate into vesicles, entrapping the aqueous solution and whatever is contained in it. Microfluidics additionally exploits the properties of flow through narrow channels confined to microscale dimensions in which diffusive mixing is significant and turbulent flow is minimized. The narrow channels and limited residence time result in unilamellar as opposed to multilamellar vesicles. The laminar flow eliminates most of the chaotic mixing, resulting in predictable and reproducible flow patterns that may be accurately characterized and modeled mathematically. The reproducibility afforded by this strategy is unachievable by conventional synthesis methods. In terms of production, this use is extended by eliminating the need for subsequent sample postprocessing such as sonication, extrusion, and freeze-thawing, all of which consume time, require additional equipment, and perhaps most importantly compromise membrane integrity and involve destruction and reformation of the vesicles [33].

#### 3.2.2. Size and Dispersity

We first determined optimal conditions for NV and DNV synthesis using fluorescein isothiocyanate (FITC) as an aqueous payload. The diameters of NVs and DNVs generated by the microfluidic methods we used here and loaded with FITC are reported in Table 1 as nanometer (nm) mean +/− standard error of the mean (sem). In all cases, NV formulations were quite homogenous in size, consistently showing polydispersity index values of 0.1 to 0.2. Regarding trends in microfluidic parameters and resultant size, we found that increasing the flow rate ratio (FRR) resulted in smaller vesicle sizes. Additionally, for fixed input parameters, smaller volume microfluidic chips produced smaller vesicles. Reducing the lipid mixture concentration in the organic stream additionally lowered the vesicle size.

NV size is perhaps the single most important factor for effective drug delivery by many routes [34, 35] and this is especially the case for transdermal delivery [36, 37]. As mentioned earlier, the limiting factor to NV-based transdermal drug delivery is transport across the stratum corneum, the main functional barrier and the outermost layer of the epidermis. The stratum corneum is formed of tightly packed corneocytes (nonviable keratinocytes that have reached terminal differentiation) which are embedded in membranous extracellular lipid structures. As protein bridges called corneodesmosomes between corneocytes degrade, intercellular pathways are formed in the stratum corneum [38, 39], which are on the order of 50 nm in diameter [40]. This extracellular pathway allows ingress through the stratum corneum. The transfollicular pathway comprising the space around the hair shaft is another available route, though this path does not reliably provide for delivery of significant amounts of therapeutic. Conventional NVs are unable to cross through the pores, rupturing as they squeeze through and leaking out payload; very small NVs of less than 50 nm have, however, been shown to have moderate success. NVs at and under this size are quite difficult to produce in sufficient quantity and have limited drug-loading capacity. However, DNV formulations, even at sizes greater than 200 nm, are able to squeeze through the lacunar domains, maintain integrity and payload, and reach the target site.

#### 3.2.3. Zeta Potential

The zeta potential of an NV is its overall charge in a particular vehicle and, in addition to size, has a great influence on particle penetrance [36]. Zeta potential has implications in passive targeting of the NVs, as well as NV stability in solution. The measured zeta potentials are reported in Table 1 as mean ± sem. In addition, we compared positively charged DOTAP to two other lipids: DCP which is negatively charged and DOPE, which is neutral. The zeta potentials were +41.7 ± 3.7 mV (DOTAP), −23.9 ± 5.6 mV (DCP), and +7.2 ± 1.9 mV (DOPE) for DNVs, showing the tunable nature of vesicle charge by incorporation of different phospholipids.

Zeta potential plays a role in the colloidal stability of NVs: a sufficiently high zeta potential (reported in some studies as ±30 mV) allows NVs to repel each other in solution and thus prevent or slow down the rate of flocculation and aggregation [41, 42]. In addition, the development of NVs and DNVs with different zeta potentials should expand the clinical application to a wide range of tissues with various cell surface charges.

### 3.3. The Modified Microfluidic Methods Significantly Improved NV/DNV Drug Encapsulation

NVs/DNVs are formed upon mixing of organic components and aqueous solution, typically by utilizing a relatively fast-flowing (1000 *μ*L/min) aqueous stream and a slower flowing (10 *μ*L/min) organic stream containing the lipid components dissolved in isopropyl alcohol (IPA) to give a high FRR of 100. This is suitable for the encapsulation of hydrophobic drugs, which accompany the membrane components in the organic stream and are thus easily embedded in the membrane upon vesicle formation when the streams mix. Unfortunately, for hydrophilic drugs, this method is largely ineffective as indicated by the low entrapment efficiency presented in Table 1. Hydrophilic drugs that exhibit good solubility in aqueous solvent must be dissolved in the fast-flowing aqueous solution loop; as a result, during the rapid mixing and vesicle formation process, most the drug does not have a chance to become entrapped within the aqueous core of the NVs and a majority of drug flows past the lipids and into the collection tube unencapsulated.

To overcome this limitation, our group developed a modified microfluidic synthesis strategy to encapsulate hydrophilic drugs. We considered that using an aqueous stream moving at a speed 5 times faster relative to the organic stream instead of a speed 100 times faster would result in a dramatically higher amount of hydrophilic drug being entrapped. Though this would result in the mix solution containing 16.7% IPA as opposed to the 1% IPA in the standard case, we surmised that this would not adversely affect NV formation, as other existing techniques, such as the reverse phase ethanol evaporation technique, are quite successful in NV production despite the large initial quantities of ethanol [43]. Realizing that the reduction of FRR in the new method would potentially sacrifice homogeneity and increase the size and dispersity of the resulting population, we rationalized that ramping up the absolute speeds of both the aqueous and organic streams while keeping the FRR fixed at 5 could rescue some of the deleterious effects of a lower FRR. For this method, we tested various low FRRs and absolute speeds and settled on an optimum of the lipid-containing IPA stream moving at 1000 *μ*L/min and the aqueous stream moving at 5000 *μ*L/min. The characteristics of DNVs produced by the standard method and the modified method are compared in Table 1. As expected, the populations synthesized through the modified method show a modest increase in size and dispersity, a small price to pay for much greater entrapment efficiency. An additional benefit of the modified method is the vast reduction in total reaction time and increase in resultant DNV concentration, which may be favorable in scaling up production.

A similar outcome to the modified method in terms of hydrophilic drug entrapment could potentially be obtained by the addition of a third inlet stream, with a slow-flowing aqueous solution of the hydrophilic drug meeting the other two streams within the reactor channels.

### 3.4. *In Vivo* Testing of Transdermal Delivery of AF647-Zol-Loaded NVs and DNVs

To demonstrate transdermal delivery and localized payload release, we selected the mouse head skin as the target transdermal tissue, closely overlaying the calvarial bone. We strategized to entrap a fluorescently labeled bisphosphonate (AF647-Zol), which has a significant affinity to mineralized tissue and would be stably absorbed on the surface of bone. The postulated transdermal penetration and the local payload release could be monitored by the fluorescent signal of AF647-Zol adsorbed on the surface of calvarial bone.

#### 3.4.1. Synthesis of AF647-Zol Loaded NVs and DNVs

**Figure 1:**
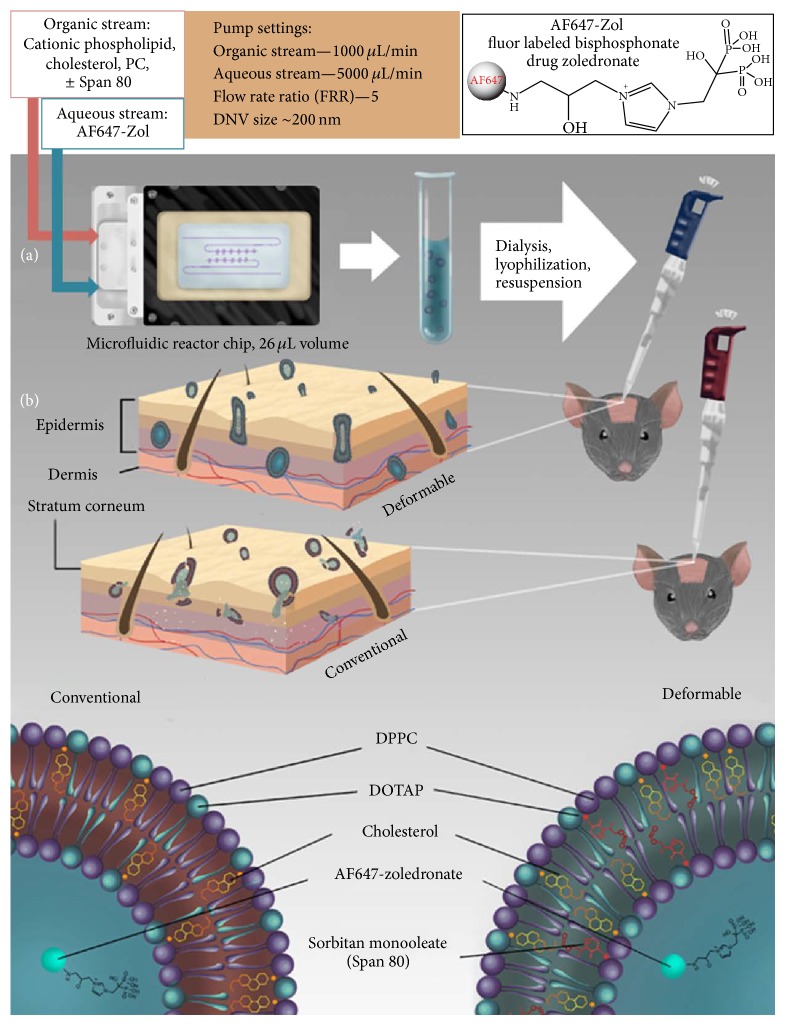
*Synthesis and testing of drug-loaded conventional and deformable NVs*. Synthesis took place in a 26 *μ*L volume microfluidic chamber, wherein controlled and confined laminar diffusive mixing of aqueous AF647-Zol (upper right, 1(a)) solution and lipid membrane components dissolved in isopropyl alcohol occurs within the microchannels and results in the formation of homogenous, reproducible populations of drug-loaded NVs, which may be made deformable by addition of the edge activator sorbitan monooleate (Span 80). The drug-loaded NVs and DNVs underwent dialysis, lyophilization, and resuspension before application to shaved calvarial skin of wildtype C57Bl6J mice. Aqueous solution of free AF647-Zol was also used. Deformable NVs more readily penetrate the outer dermis (upper dermal image) through nanopores without rupturing which may occur with conventional NVs (lower image, 1(b)) and releasing cargo before reaching the target.

The reactor-based modified method described above was used to synthesize NVs and DNVs encapsulating AF647-Zol for the purpose of determining localized transdermal delivery to underlying bone. Since skin layers are known to be negatively charged [44], positively charged vesicles have been shown to improve penetration and retention in the skin [45–47]. It is therefore theorized that NVs as well as DNVs having a positive surface charge may fare better in terms of penetration into and retention in the skin as compared to similar neutral or negatively charged NVs [48, 49]. For this project, we synthesized positively charged NVs and DNVs (Figure 1(a)). The overall schematic for production and application of AF647-Zol NV/DNV (and unencapsulated aqueous AF647-Zol) is shown in Figure 1(b). The reactor-produced NVs and DNVs were lyophilized and stored before use.

#### 3.4.2. Stability

**Figure 2:**
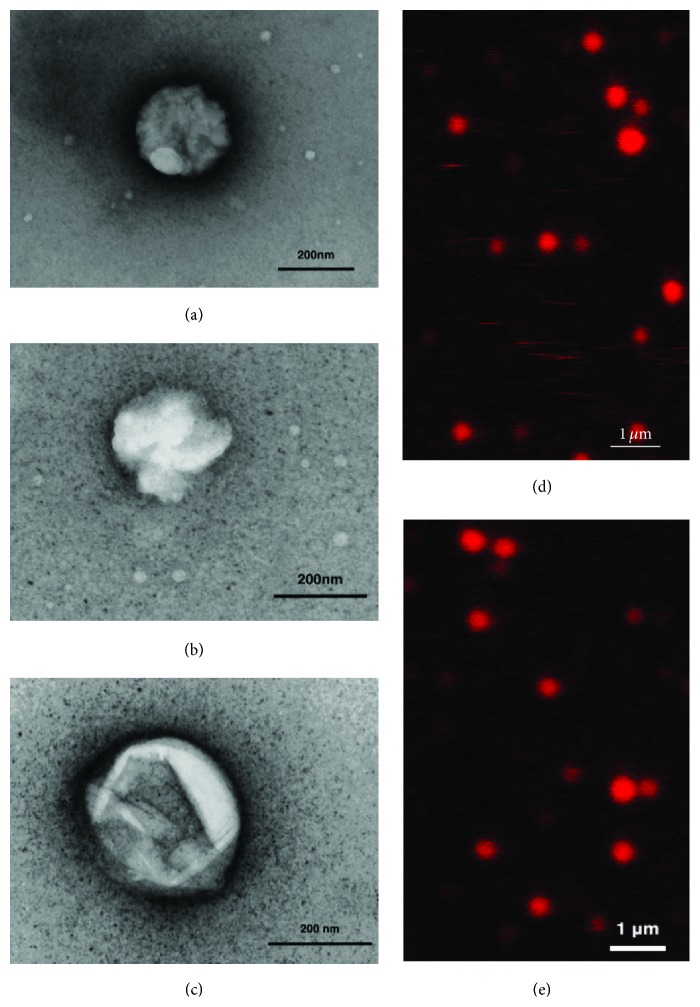
*Transmission electron and confocal micrographs of AF647-Zol-loaded NV/DNVs synthesized in a microfluidic reactor.* Shown are (a) a conventional NV and (b) an AF647-Zol-loaded DNV, both produced by our modified microfluidic method, viewed at 72,000x. In (c), an AF647-Zol-loaded DNV produced by the standard microfluidic method that underwent lyophilization, storage at 277 K, and resuspension produced by the regular microfluidic method is viewed at 100,000x. Images were obtained using a uranyl acetate stain and copper mesh background on a JEOL 100CX transmission electron microscope. Estimated diameters are (a) 246 nm, (b) 241 nm, and (c) 298 nm. Colloidal solutions of AF647-Zol-loaded NVs and DNVs in (d) and (e), respectively, were imaged by confocal laser microscopy using a Leica TCS SP5 microscope following dialysis, lyophilization, and resuspension. The largest DNV depicted is estimated to be ~400 nm in diameter, and the vesicles are quite homogenous in size. The fluorescent drug is contained only within the NVs and DNVs and is not in solution, confirming stability.

The ability of reactor-produced NV/DNVs to withstand processing techniques for storage, primarily lyophilization, is critical in determining their shelf life, pharmaceutical utility, and transportability. Our investigations suggest that DNVs have long term stability after lyophilization and indeed show minimal drug leakage, as shown in Figure 2(d). DNVs loaded with AF647-Zol synthesized by the modified microfluidic method were subject to dialysis to remove free drug from solution and then lyophilized to a powder. Two weeks following resuspension in deionized water, they were imaged by confocal microscopy. The images clearly show that the fluorescence is contained only in the spherical vesicle structures and not found diffusely in solution suggesting that the membrane integrity was not compromised during the lyophilization and resuspension process and that drug leakage did not occur. Furthermore, confocal microscopy indicates the sizes after lyophilization correlate quite well with initial measurements obtained prior to lyophilization through dynamic light scattering (Table 2).

#### 3.4.3. Characteristics of AF647-Zol NV/DNVs

As shown in Table 2, the size and zeta potential were very similar to the NV/DNVs encapsulating FITC in the prestudy method development.

#### 3.4.4. Morphology and Deformability

The morphology of microfluidic-produced AF647-Zol-loaded NV/DNVs was studied through fixation, negative staining, and transmission electron microscopy. Representative micrographs of 500x diluted, drug-loaded samples out of the reactor chip are presented in Figures 2(a), 2(b), and 2(c). The images show spherical and globular vesicle structures for NV/DNVs synthesized by a standard method and DNVs synthesized by a modified method. NV/DNVs were roughly 240 nm in size. This size measured from electron microscopy differs from that obtained by dynamic light scattering not only because the techniques utilize different principles but also because electron microscopy requires fixation, adherence, and drying of the vesicles on a membrane, which flattens them and may artificially increase size.

**Figure 3:**
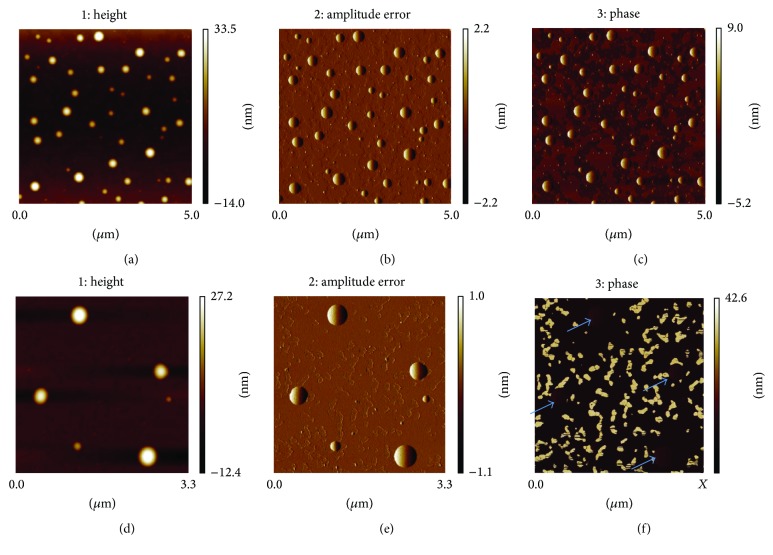
*Atomic force microscopy of AF647-Zol-loaded NVs and DNVs*. The height, amplitude error, and phase are shown for conventional NVs ((a), (b), and (c), resp.) and DNVs ((d), (e), and (f), resp.). The higher phase contrast in DNVs in (f) indicated by blue arrows compared to conventional NVs (c) suggests higher deformability of the vesicles under the same applied imaging force.

The atomic force microscopic (AFM) analysis revealed the similar uniform spherical morphology of AF647-Zol-loaded NVs and DNVs in the height images (Figures 3(a) and 3(d), resp.) as well as the amplitude error images (Figures 3(b) and 3(e), resp.). However, the phase image of AF647-Zol-loaded NVs and DNVs differs significantly (Figures 3(c) and 3(f), resp.). Phase imaging monitoring the phase lag between signals can be used to map variations in surface properties such as elasticity. While quantitative measurements were not obtained, the deformable features of DNVs as compared to NVs were clearly indicated. The higher phase contrast observed in phase imaging of the DNVs (Figure 3(f)) as compared to conventional NVs indicates increased deformability of the nanovesicles.

#### 3.4.5. Transdermal Delivery of AF-Zol Bisphosphonate and Tissue Distribution

**Figure 4:**
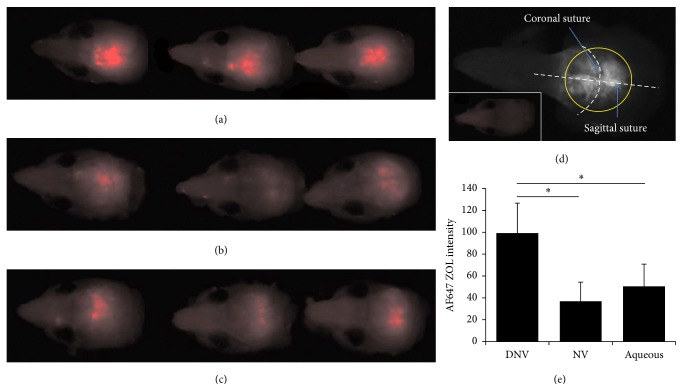
*AF647-Zol calvarial bone distribution*. The AF647-Zol signal is the greatest with DNV delivery (a) of the 3 methods used. The signal is reduced with either conventional NV delivery (b) or delivery of free aqueous AF647-Zol (c); in fact aqueous delivery appears to be slightly more effective than NV delivery. The skull anatomy is shown with the region of interest (ROI) circled (d). AF647 signal levels in the ROI were quantified (e), revealing DNV delivery is significantly (^*∗*^*p* < 0.05) greater than either NV or aqueous delivery.

The proof-of-concept demonstration of the enhanced capability of the microfluidic-produced DNVs to deliver a drug payload transdermally compared to the NVs or vehicle was done by application of the vesicles in a paste to the shaved head skin of mice. After transdermal application, the fluorescent AF647-Zol signal from the target tissue—calvarial bone—was the greatest for DNVs (Figure 4(a)) compared to the NVs (Figure 4(b)) and the application of AF647-Zol aqueous solution (Figure 4(c)). The anatomy of the calvarial area is shown in Figure 4(d), and the signals quantified are shown in Figure 4(e), revealing the increase with AF647-Zol-DNV to be significant (*p* < 0.05).

The DNVs, therefore, had the greatest success transversing the stratum corneum and remaining skin layers and delivering the drug to the bone. DNVs also provided the most consistent results, in terms of degree of staining, which was very similar for all mice in that group. Cryosectioned calvarial bone counterstained with DAPI (labeling nuclei) and imaged by confocal laser scanning microscopy (CLSM) showed the uniform AF647-Zol signal on the external bone surface interfacing the dermal tissue for DNV delivery (Figure S3C in Supplementary Material available online at https://doi.org/10.1155/2017/4759839), suggesting the local payload release within the dermal tissue.

#### 3.4.6. Subcutaneous Localization

The luminescent analyzer images of the outer surface of the calvarial skin, where the formulations were applied, are presented in Figure S2. To further understand the fate of the drug in the skin, we cryosectioned the excised calvarial skin, stained with DAPI, and examined the samples through CLSM. Both the DNVs and NVs (A, B) show notable follicular affinity, particularly as compared to the aqueous solution (C). DNVs show the highest retention within the skin layers, which may indicate increased skin adherence and thus resistance against rodent grooming and licking behavior. Two forces that may contribute to rupture of NVs and DNVs to release their drug payload in the deeper epidermal and dermal layers are the osmotic pressure gradient and interactions with the embedded lipid membranes and subcutaneous fat. Our data suggests that DNVs withstand these forces better than NVs.

#### 3.4.7. Systemic Leakage

AF647-Zol is a bone-targeting drug that labels any bone (specifically, calcium depot) with which it comes in contact. Thus femur bones were collected from mice in* in vivo* trials and imaged to assess the degree of systemic leakage resulting from each applied formulation. These results, presented in Figure S1, show that there is some degree of systemic drug leakage when the aqueous solution is applied, which is less when the drug is encapsulated in DNVs.

## 4. Conclusion

In this series of investigations, we have presented the development of an adaptable transdermal drug delivery system based on microfluidic-produced DNV technology. Lipid nanoparticle technology, which is well-characterized and mature, may at long last realize its full potential via the enabling microfluidic platform which overcomes a multitude of existing barriers to reproducibility, tunability for size, and scalable synthesis and has the potential to pave the way for the next generation of nanovesicle-based pharmaceuticals to reach the clinic. To enable loading of various drug classes and polarities and thus expand the utility of this drug delivery system, we developed a modified microfluidic method to overcome technical challenges in encapsulating significant quantities of hydrophilic molecules by altering the flow speeds and relative flow ratios of the component-containing streams fed into the reactor chip.

In a murine model, we have shown proof-of-concept for transdermal drug delivery using the microfluidic-produced DNVs carrying the bisphosphonate drug zoledronate which outperform conventional NVs and aqueous drug solutions in delivering payload to underlying bone.

The tunability of the DNV size in the microfluidic system could enable expanded utility of the DNVs from transdermal to topical drug delivery systems. By slowing down the flow speeds and increasing DNV size, larger DNVs can be generated. Larger DNVs tend to aggregate and be retained in the epidermal and dermal layers, releasing their payload upon fusion with the extracellular lipid membranes present in those layers. A similar strategy may enable the technology to be used successfully in transmucosal delivery. Transport through mucosal membranes presents similar advantages to transdermal delivery but requires different features: larger vehicles with a high molecular weight and high charge to resist being washed away and be more effectively retained in the mucosa.

While the current microfluidic system offers a number of remarkably useful advantages suitable for large scale production of DNVs, we believe that we can continue to optimize the synthesis conditions such as mixing of the streams, which is currently confined to diffusion. We will continue to explore temperature and pressure changes and possibly use acoustic energy applied to the microfluidic reactor as part of this process. The eventual goal is to encapsulate hydrophobic as well as hydrophilic drugs and thus expand the therapeutic landscape of candidate molecules that could be used for human disorders.

## Supplementary Material


**Supplementary Figure 1**. *AF647-Zol in skin and femur*. The AF647-Zol signal is shown in calvarial skin from a non-treated control (A) and 3 mice each receiving drug by DNV (B), NV (C), or aqueous solution (D). In 2 of 3 mice, the signal is lowest for DNVs as compared to other groups (excluding control). Signal from femur reflects distribution beyond the target tissue (skull under the application site). A femur from an untreated mouse (E) and from mice treated by DNV (F), NV (G), and aqueous solution (H) reveal slightly greater signal intensity in femur heads in G and H as compared to F.
**Supplementary Figure 2**. *AF647-Zol signal in transected skin and bone*. The AF647-Zol signal in cryosectioned bone and overlying dermis imaged by confocal microscopy from mice receiving drug via DNVs (A), NVs (B), and by aqueous solution (C) is shown, revealing great mouse-to-mouse variability.
**Supplementary Figure 3**. *Cross-section of calvarial bone*. In confocal representative images of cryosectioned calvarial (skull) bone, the signal for AF647-Zol was not apparent in mice treatment by aqueous solution (A), but some surface signal could be seen with NV delivery (B), the greatest signal was seen with DNV delivery (C).

## Figures and Tables

**Table 1:** *Characteristics of FITC-loaded DNVs/NVs generated by the standard and modified methods*. DNVs loaded with FITC were synthesized using a standard microfluidic method using a relatively fast aqueous flow rate (1000 *μ*L/min) and slow organic flow rate (10 *μ*L/min) and thus a FRR of 100. In the modified method, the aqueous pump rate was to 5000 *μ*L/min and organic 1000 *μ*L/min, for a resulting FRR of 5. Conventional (nondeformable) NVs were synthesized by the standard method. The resultant diameters by either method or type of NVs (deformable; nondeformable) were similar, with NVs at 196 ± 39 nm, DNVs by the standard method at 202 ± 17 nm, and DNVs by the modified method at 221 ± 41 nm. Zeta potentials were also similar with DNV standard > DNV modified > NV standard. Entrapment efficiency was greatly impacted by method for DNVs, with efficiency being much higher with the modified method for DNVs; entrapment efficiency was similar for modified-method DNVs and conventional NVs.

Nanovesicle (NV) type	DNVs synthesized by standard microfluidic method (low IPA speed; high FRR)	DNVs synthesized by modified microfluidic method (high speed; low FRR)	Conventional NVs
*Characteristics*			
Size (diameter)	202 ± 17 nm	221 ± 40 nm	196 ± 39 nm
Zeta potential	46 ± 2.4 mV	41.7 ± 3.7 mV	38.1 ± 1.8 mV
Entrapment efficiency (FITC dye)	1.1%	39.6%	37.4%

**Table 2:** *Characteristics of AF647-Zol-loaded DNVs/NVs*. AF647-loaded NVs and DNVs were prepared in the microfluidic reactor using the modified method. DNVs were slightly larger in diameter than NVs and also had a greater zeta potential.

Type	DNVs	Conventional NVs
*Characteristics*		
Size (diameter)	236 ± 62 nm	204 ± 47 nm
Zeta potential	33.8 ± 3.5 mV	29.2 ± 1.4 mV
