# Supplementary figures and images for: Deformable Nanovesicles Synthesized through an Adaptable Microfluidic Platform for Enhanced Localized Transdermal Drug Delivery
Source: J Drug Deliv. 2017 Apr 5;2017:4759839. doi: 10.1155/2017/4759839 (PMC5396447; doi:10.1155/2017/4759839)

Supplementary Figures

Supplementary Figure 1.

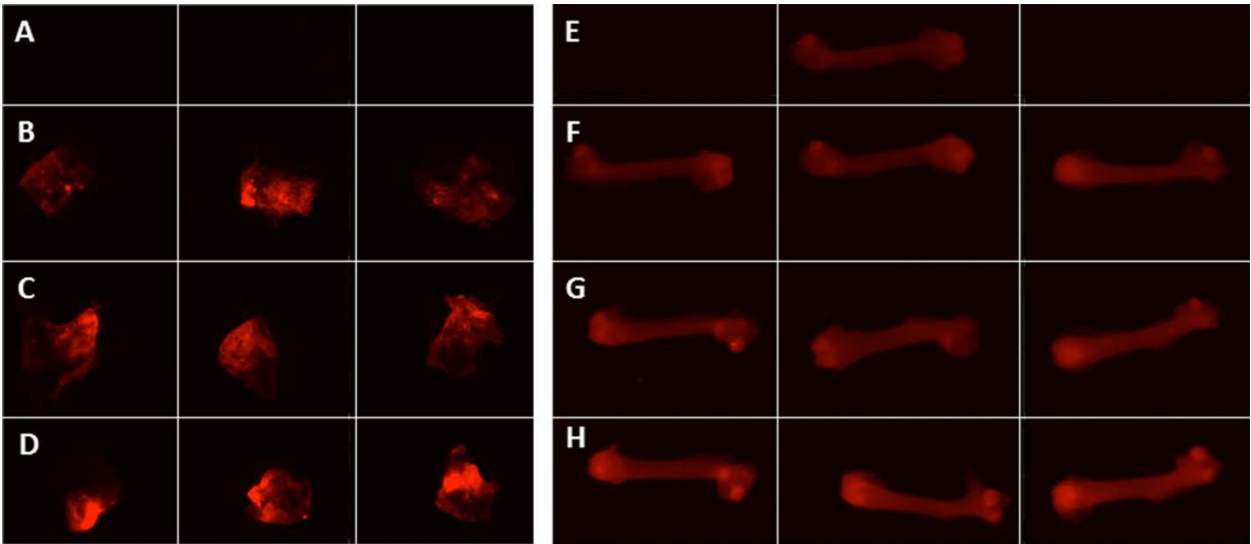

Supplementary Figure 2.

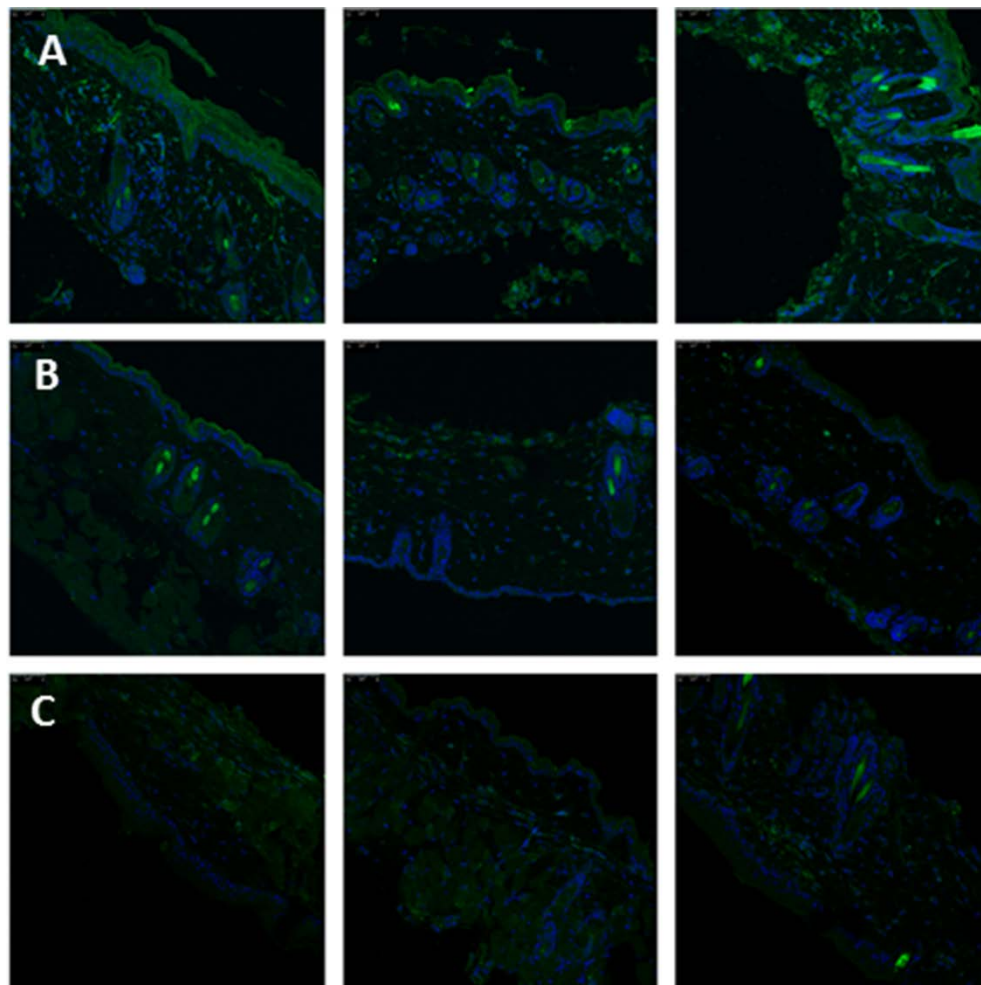

**Supplementary Figure 3**

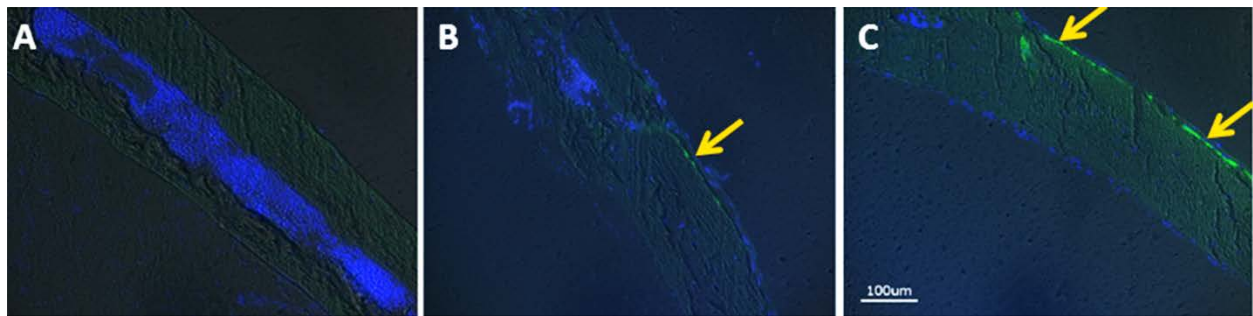

Supplement: Supplementary file 1 — Supplementary Figure 1. AF647-Zol in skin and femur. The AF647-Zol signal is shown in calvarial skin from a non-treated control (A) and 3 mice each receiving drug by DNV (B), NV (C), or aqueous solution (D). In 2 of 3 mice, the signal is lowest for DNVs as compared to other groups (excluding control). Signal from femur reflects distribution beyond the target tissue (skull under the application site). A femur from an untreated mouse (E) and from mice treated by DNV (F), NV (G), and aqueous solution (H) reveal slightly greater signal intensity in femur heads in G and H as compared to F. Supplementary Figure 2. AF647-Zol signal in transected skin and bone. The AF647-Zol signal in cryosectioned bone and overlying dermis imaged by confocal microscopy from mice receiving drug via DNVs (A), NVs (B), and by aqueous solution (C) is shown, revealing great mouse-to-mouse variability. Supplementary Figure 3. Cross-section of calvarial bone. In confocal representative images of cryosectioned calvarial (skull) bone, the signal for AF647-Zol was not apparent in mice treatment by aqueous solution (A), but some surface signal could be seen with NV delivery (B), the greatest signal was seen with DNV delivery (C). [file 4759839.f1.pdf]
